# Oxidative stress in pituitary neuroendocrine tumors: Affecting the tumor microenvironment and becoming a new target for pituitary neuroendocrine tumor therapy

**DOI:** 10.1111/cns.14315

**Published:** 2023-06-21

**Authors:** Yuhang Zhou, Anke Zhang, Chaoyou Fang, Ling Yuan, Anwen Shao, Yuanzhi Xu, Danyang Zhou

**Affiliations:** ^1^ The First Clinical Medical College Heilongjiang University of Chinese Medicine Harbin China; ^2^ Health Management Center Tongde Hospital of Zhejiang Province Hangzhou China; ^3^ Department of Neurosurgery, The Second Affiliated Hospital, School of Medicine Zhejiang University Hangzhou China; ^4^ Department of Neurosurgery, Shanghai General Hospital, School of Medicine Shanghai Jiao Tong University Shanghai China; ^5^ School of Public Health, School of Medicine Shanghai Jiaotong University Shanghai China; ^6^ Department of Neurosurgery, Huashan Hospital, School of Medicine Fudan University Shanghai China

**Keywords:** immunotherapy, oxidative stress, pituitary adenoma, pituitary neuroendocrine tumors, tumor microenvironment

## Abstract

Pituitary adenomas (PAs), or pituitary neuroendocrine tumors (PitNETs), are commonly found in the anterior pituitary gland. Although the majority of PitNETs are benign and stable, several tumors have malignant characteristics. The tumor microenvironment (TME) plays an important role in the process of tumorigenesis and is composed of several types of cells. Various cells in the TME are significantly affected by oxidative stress. It has been reported that immunotherapeutic strategies have good effects in several cancers. However, the clinical potential of immunotherapies in PitNETs has not yet been fully discussed. Oxidative stress can regulate PitNET cells and immune cells in the TME, thus affecting the immune status of the TME of PitNETs. Therefore, modulation of oxidative stress‐regulated immune cells using a combination of several agents and the immune system to suppress PitNETs is a promising therapeutic direction. In this review, we systematically analyzed the oxidative stress process within PitNET cells and various immune cells to elucidate the potential value of immunotherapy.

## Graphical abstract

In this study, we briefly reviewed the mechanism of oxidative stress‐mediated immunotherapy in pituitary neuroendocrine tumors (PitNETs). In addition, the experimental research on oxidative stress‐related immunotherapy in PitNETs was comprehensively summarized, which may be the basis for determining better treatment methods in the future.

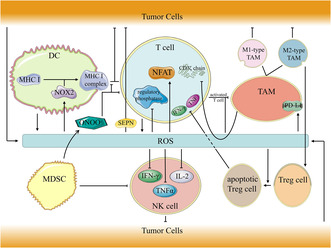

## 1 INTRODUCTION

Pituitary adenomas (PAs), recently named pituitary neuroendocrine tumors (PitNETs) according to the new classification and nomenclature established by WHO 2022, are commonly found in the anterior pituitary gland and are harmful to the endocrine system and human health.^1^ It has been suggested that the prevalence of PitNETs is approximately 20%; however, most tumors present no clinical manifestation.^2^, ^3^, ^4^ Additionally, the incidence of clinically significant PitNETs is 80–100 cases per 100,000 people.^2^, ^5^ PitNETs are generally classified as benign (~65%), invasive (~35%) or malignant (only ~0.2%), or as macroadenomas (≥10 mm) and microadenomas (<10 mm) according to their size.^1^, ^6^, ^7^ Although most PitNETs are stable benign tumors with a good clinical prognosis after surgical excision, a minority of PitNETs are still difficult to control, even with advanced drugs and radiotherapy, so‐called aggressive tumors.^8^, ^9^ Therefore, the underlying mechanisms and microenvironments involved in the development of PitNETs require further investigation. Similar to other tumors, factors such as cell cycle dysregulation, abnormal metabolic pathways, growth factor overexpression, defective signaling pathways, epigenetic silencing of tumor suppressor genes, oncogene overexpression, and an altered microenvironment are the focus of research attention in PitNETs.^10^, ^11^, ^12^ The transformation of normal cells into tumor cells is accompanied by changes in cellular metabolism and mitochondrial function, which means that the internal physiological responses of tumor cells are usually significantly different from the external effects of normal cells.^13^, ^14^ Moreover, transformed cells can recruit and corrupt other cells to establish the tumor microenvironment (TME).^15^ However, there are few studies on the composition of the PitNET microenvironment and how it regulates PitNET progression.

Oxidative stress usually occurs when intracellular reactive oxygen species (ROS) levels exceed the cellular antioxidant capacity, resulting in an imbalance in oxidative homeostasis.^16^ Numerous studies have confirmed the relationship between oxidative stress and the formation and progression of various human pathologies.^17^, ^18^ Intracellular ROS are mainly generated during mitochondrial oxidative phosphorylation and play a crucial role in cellular signaling pathways, but excessive ROS damage genomic and mitochondrial DNA, leading to DNA damage, molecular mutations, and altered signaling pathways.^16^, ^19^ Tumor cells often have abnormal alterations in metabolic pathways and mitochondrial function, which lead to the production of ROS and oxidative stress within tumor cells and further affect tumorigenesis.^20^ However, the process of oxidative stress and its role in PitNETs have not been fully discussed.

The importance of the TME as a therapeutic target and prognostic factor in tumor treatment has been widely recognized.^21^, ^22^ Multiple factors in the TME can have an impact on the progression and persistence of tumors; the effects of immunotherapy (IMT), a central part of current cancer treatment, are strongly associated with the TME.^23^ Reactive oxygen species are important signaling mediators for multiple physiological processes in the TME and for the immune cells within it.^24^, ^25^ Therefore, the interaction between oxidative stress and the TME is considered of great importance for tumor treatment. This review focuses on the effects of oxidative stress and the TME in PitNETs and discusses novel therapeutic strategies based on the modulatory role of oxidative stress in the TME that can be used to treat PitNETs.

## 2 OVERVIEW OF THE OXIDATIVE STRESS RESPONSE IN TUMORS

Oxidative stress occurs when the balance between ROS and the antioxidant system is disrupted, and it is mainly generated by mitochondria.^20^ In addition, inflammatory cells, such as neutrophils, macrophages, and eosinophils, generate exogenous ROS through NADPH oxidase reactions. Excessive ROS will destroy and/or change DNA, RNA, lipids, proteins, and other intracellular molecules.^26^ Correspondingly, a cytokinesis‐block micronucleus cytome (CBMN‐cyt) assay showed that the frequency of biomarkers of chromosome breakage and/or loss, biomarkers of DNA misrepair and/or telomere end‐fusions, and biomarkers of elimination of amplified DNA and/or DNA repair complexes were significantly increased in PitNET patients.^27^, ^28^, ^29^ The researchers believe that these findings indicate that chromosome/oxidative DNA damage is related to the invasion of PitNETs.^29^ Furthermore, ionizing radiation and some environmental factors participate in all stages of carcinogenesis induced by ROS.^20^ PitNETs show increased ROS levels and signs of oxidative damage, and the secretion of a large amount of ROS/reactive nitrogen species (RNS) recruits more activated immune cells, magnifies the dysregulated processes and eventually leads to a preneoplastic condition.^14^, ^20^ When entering a state of oxidative stress, irreversible oxidative damage to DNA, RNA, lipids and proteins may lead to genetic changes, thus leading to the imbalance of oncogenes and tumor suppressor genes.^30^ These physiological changes indicate that oxidative stress plays an important role in the development of PitNETs. Notably, although the ROS level has increased to a certain extent, tumor cells can induce a new redox balance so that they can adapt and proliferate, which is a significant feature of cancer cells that is different from normal cells.^30^ In fact, pituitary cancer and PitNET are still difficult to distinguish in terms of pathology, immunohistochemistry, genetic analysis, and ultrastructural imaging findings.^31^ Therefore, an understanding of oxidative stress‐related pathways may aid in the identification of pituitary cancers and the treatment of PitNETs.

The oxidative stress pathway is very complex. In addition to the mainstream pathway, there are some special pathways associated with PitNETs. The Ras pathway is an important pathway related to oxidative stress and cancer. Approximately 30% of human tumors contain activated mutations of Ras family oncogenes, and these mutations cause the proteins to have constitutive activity.^32^, ^33^ Overexpression of the Ras family causes increased mitochondrial mass and ROS accumulation, leading to DNA damage and promoting transformation.^34^ The Ras pathway relies on the NADPH oxidase Nox4.^34^ Ras mutation has been proven to be related to human pituitary tumors, and H‐ras point mutation has been found in distant metastatic pituitary cancer, which indicates that H‐ras gene mutation may play an important role in the formation of pituitary cancers and the metastasis of PitNETs.^31^, ^35^, ^36^ In addition, studies have shown that the epidermal growth factor receptor (EGFR) signaling pathway, including nuclear factor erythroid 2‐related factor 2 (Nrf2), mitogen‐activated protein kinase ERK1/2, MEK and protein kinase C, is related to cell proliferation and affected by oxidative stress.^37^, ^38^ Multiomics studies on PitNETs have revealed significant changes in the Nrf2‐mediated oxidative stress response pathway and the corresponding regulatory factors.^39^, ^40^ Nrf2 regulates the expression of hundreds of genes, including those related to antioxidant enzymes, tissue remodeling and fibrosis, immune and inflammatory reactions, carcinogenesis and metastasis, which can regulate ROS levels together with Kelch‐like ECH‐associated protein 1 (Keap1).^41^ Nrf2 can also regulate cell protective enzymes such as glutathione peroxidase (GPx) and oxidoreductase to combat oxidative stress.^42^, ^43^ In general, Nrf2 plays a central role in the oxidative stress‐related pathways of tumors, including PitNETs.

## 3 ALTERED OXIDATIVE STRESS‐RELATED PATHWAYS IN PITNETS

### 3.1 Nrf2‐mediated oxidative stress response pathway

Nrf2 is a key regulator of oxidative stress, and it interacts with Keap1 to induce an antistress response.^44^ Previous studies have confirmed that Nrf2 expression is increased in PitNETs, and the level of Nrf2 phosphorylation is also increased.^14^ Increased ROS levels activate the Nrf2‐Keap1 complex in the cytoplasm through multiple interacting signaling pathways.^45^, ^46^, ^47^ In the presence of oxidants, the DGR structural domain of Keap1 releases the DLG motif of Nrf2, thereby preventing ubiquitination and degradation of Nrf2.^44^, ^48^, ^49^, ^50^, ^51^ Upon activation, isolated and phosphorylated Nrf2 rapidly enters the nucleus to interact with antioxidant response elements (AREs).^52^, ^53^, ^54^ This reduces oxidative damage by inducing the synthesis of antioxidant proteins^54^, ^55^, ^56^ but also promotes tumorigenesis by regulating cellular metabolism.^54^, ^57^ Antioxidant genes such as heme oxygenase 1 (HO‐1) contain upstream ARE sequences, which is an important reason why Nrf2 is considered to have a central position in the oxidative stress pathway.^44^, ^58^, ^59^

The role of the Nrf2 signaling pathway in PitNETs has been verified. Irradiation‐treated C57/BL6 mice exhibited oxidative damage due to Nrf2 activation and HO‐1 expression, which could be inhibited by the antioxidant agent pituitary adenylate cyclase‐activating polypeptide 38 (PACAP38).^54^, ^60^ A study found that patients with PitNETs had significantly more chromosomal and oxidative DNA damage than normal subjects and that oxidative stress was an important cause of this phenomenon.^29^ Similar to other tumors, the occurrence and development of PitNETs are closely related to oxidative stress.^44^, ^54^ Thus, Nrf2, which is at the core of the response, plays a broad and critical role in PitNET and may be a new target for PitNET treatment.

### 3.2 Mitochondrial dysfunction pathways

Studies have shown that mitochondrial dysfunction is significantly associated with the pathogenesis of PitNETs.^39^, ^61^ Mitochondria, as a source of ROS, are the center of oxidative stress and the main location of energy metabolism.^62^ Altered energy metabolism has been recognized as a marker of tumors, and such alterations may lead to increased ROS production.^63^ Accordingly, enlargement of the mitochondrial area can be detected in the early stages of PitNET, and significantly increased expression of major factors that regulate mitochondrial gene expression, such as Nrf1, Nrf2, and mitochondrial transcription factor A (TFAM), was found and maintained.^14^, ^64^

Mitochondrial fusion and fission processes regulate mitochondrial morphology, and the expression of the fusion‐related kinetic proteins mitofusin 2 (MFN2) and optic atrophy type 1 (OPA1) was found to be enhanced with the development of PitNET, which was associated with a decrease in mitochondrial elongation and a tendency toward roundness.^14^, ^65^ In contrast, increased levels of the fission‐related protein dynamin‐related protein 1 (DRP1) indicated that mitochondrial fission was inhibited, which explains the mitochondrial morphology in PitNETs.^14^, ^65^, ^66^ An elevated rate of glycolysis is a common feature of tumor cells, and it is logical that there is also a consistent increase in lactate in PitNETs.^14^, ^67^, ^68^ All of the above findings imply the presence of significant mitochondrial dysfunction in PitNETs.

In addition, the mitochondrial OXPHOS system, which is the main generator of endogenous ROS, is usually defective in tumor cells, but OXPHOS activity is not disrupted in PitNETs.^14^, ^69^ In addition to ROS production, mitochondrial nitric oxide synthase (mtNOS) induces the production of much of nitric oxide (NO), which reacts with superoxide radicals to generate peroxynitrite anion (ONOO‐) or hydroxyl radical ·OH.^62^, ^70^ The increased rate of glycolysis and the maintenance of OXPHOS system activity may provide PitNETs with adequate ATP and raw materials to help them have a higher metabolic rate.^71^

In conclusion, mitochondrial dysfunction leads to an increase in ROS in tumor cells, which mediates relevant signaling pathways and activates procancer signaling pathways that regulate tumor progression, angiogenesis, metastasis, and survival.^62^ However, this also activates the antioxidant system of tumor cells, allowing them to survive at higher ROS levels.^72^ This endows PitNET cells with a redox system and associated signaling pathways that are different from those of normal cells.

## 4 PROFOUND ASSOCIATION BETWEEN OXIDATIVE STRESS AND THE TME

### 4.1 Overview of the TME

The TME is the environment in which tumors exist, and the significance of TME angiogenesis for tumors has been extensively studied; disorganized, leaky vessels cause an increase in interstitial pressure and a reduction in the efficiency of oxygen and nutrient delivery.^23^, ^73^ Vascular endothelial growth factor (VEGF) and its receptor (VEGFR) are central to tumor angiogenesis.^73^, ^74^, ^75^ Researchers have found that PitNETs exhibit the senescence‐associated secretory phenotype (SASP), which is regulated by NF‐κB. VEGF and matrix metalloproteinase 9, as paracrine components of SASP, may contribute to the metastasis of PitNETs.^76^, ^77^, ^78^, ^79^, ^80^ Upregulation of VEGF usually implies tumor development and malignant transformation, and this is indeed the case in PitNETs.^15^, ^81^ Compared with adjacent normal pituitary tissue, PitNET tissue shows decreased VEGF expression, while pituitary carcinoma tissues show increased expression.^82^ Furthermore, angiogenesis directly affects the oxygen and nutrient supply to the tumor, and the effects on oxygen tension and immune infiltration are also important.^23^, ^83^ This has led to the use of antiangiogenic therapy and treatment modalities in combination with immunotherapy in clinical trials.^73^, ^84^

NF‐κB‐activated proinflammatory molecules are an important method through which NF‐κB affects PitNETs.^76^ In the development of PitNETs, TNFα and IL‐1β are specific targets for NF‐κB, and they increase with the level of p‐p38 MAPK protein, which accompanies strong activation of NF‐κB.^76^, ^85^ Studies conducted in invasive PitNETs have shown that both TNFα levels and IL‐6 levels were upregulated in invasive PitNETs.^86^ They are believed to enhance the invasiveness of tumor cells and protect them from immune cell attacks.^87^, ^88^ Interestingly, some researchers have found that IL‐6 has a dual role, inhibiting PitNET growth and malignant transformation by triggering senescence.^89^, ^90^ The release of IL‐6 and IL‐8 was detected in cultured adenoma cells, which was influenced by IL‐1β dose‐dependent stimulation. Correspondingly, IL‐8 also has a similar effect to IL‐6.^91^ Another downstream factor of NF‐κB that plays an important role in the senescence of tumors is CXCR2. Knocking down CXCR2 alleviates both replicative and oncogene‐induced senescence and reduces DNA damage responses.^92^ In contrast, ectopic expression of CXCR2 leads to premature senescence via a p53‐dependent mechanism.^92^ CXCR2 is upregulated in preneoplastic lesions in vivo, and its role in promoting the invasiveness of PitNETs has also been confirmed.^92^, ^93^ In addition, TGFβ is also believed to have a regulatory effect on the development of PitNETs.^94^ As the regulatory protein of TGFβ, P27 gradually decreases during the development of pituitary tissue tumorigenesis.^95^ As expected, experiments conducted in mouse GH4 cell lines showed that TGFβ dose dependently inhibited GH4 cell proliferation.^94^ These proinflammatory molecules are important components of the TME and are closely related to NF‐κB. They participate in regulating the growth of PitNETs through various pathways.

Antagonist pituitary follicular (FS) cells are sources and targets of growth factors and cytokines,^96^ and the various factors mentioned earlier are closely related to them. In an experiment based on a nude mouse model, researchers implanted TtT/GF cells (an FS cell like mouse tubular cell line) and MtT/S cells (a rat tubular somatotrophic tubular cell line) into nude mice. Nude mice implanted with MtT/S cells alone did not form or only formed very small tumors, while nude mice implanted with TtT/GF cells at the same time generally developed large tumors.^97^ This indicates that FS cells have a promoting effect on the growth of PitNETs.^97^ Studies have found that the K_ATP_ channel is expressed in FS cells, and its activation inhibits VEGF secretion at the transcriptional level, which plays a regulatory role in the development of PitNETs.^98^ Another study based on TtT/GF cells showed that IL‐6 promotes cell proliferation in a dose‐ and time‐dependent manner under low‐density seeding conditions. For high‐density seeding, researchers believe that the high concentration of IL‐6 secreted by cultured cells themselves playes a promoting role.^99^ This indicates that FS cells also have a mechanism of autocrine growth stimulatory loop.^99^ In addition, TNFα was also found to be involved in the diffusion mechanism of inflammatory signals related to FS cells.^96^ These effects related to FS cells indicate the crucial role of FS cells in the TME of PitNETs.

The TME contains a variety of infiltrating immune cells, including tumor‐associated macrophages (TAMs), cytotoxic T cells, mast cells, and myeloid‐derived suppressor cells (MDSCs), which are thought to have antitumor effects.^22^, ^100^ Among the different subtypes of TAMs, M2‐type TAMs promote the proliferation and migration of primary tumor cell cultures more than M1‐type TAMs, exhibiting immunosuppressive characteristics.^101^, ^102^ In 80% of noninvasive tumors, the number of M2‐type TAMs was less than that of M1‐type TAMs.^102^ In addition, matrix metalloproteinase‐7 (MMP‐7) in the hypoxic region of the TME inhibited tumor cell lysis by cleaving Fas ligand, and increased expression of MMP‐7 was found to be associated with TAMs.^23^, ^103^, ^104^ These phenomena reveal the possible effects of TAMs on tumors.^15^ Myeloid‐derived suppressor cells (MDSCs) effectively inhibit proinflammatory cytokines and both CD8^+^ and CD4^+^ T‐cell activation.^105^, ^106^, ^107^ Under hypoxia, MDSCs suppress antigen‐specific and antigen‐nonspecific T cells, and the expansion of T cells is decreased, generating an immunosuppressed TME state.^23^, ^108^ This demonstrates that oxygen has an important effect on the TME, especially on the function of the immune cells therein. In this section, we summarize the effect of oxidative stress on immune cells in the TME and suggest that it might become a new target of treatment.

### 4.2 Oxidative stress regulation of the TME immune status

#### 4.2.1 Modulation of various immune cells by oxidative stress

**FIGURE 1:**
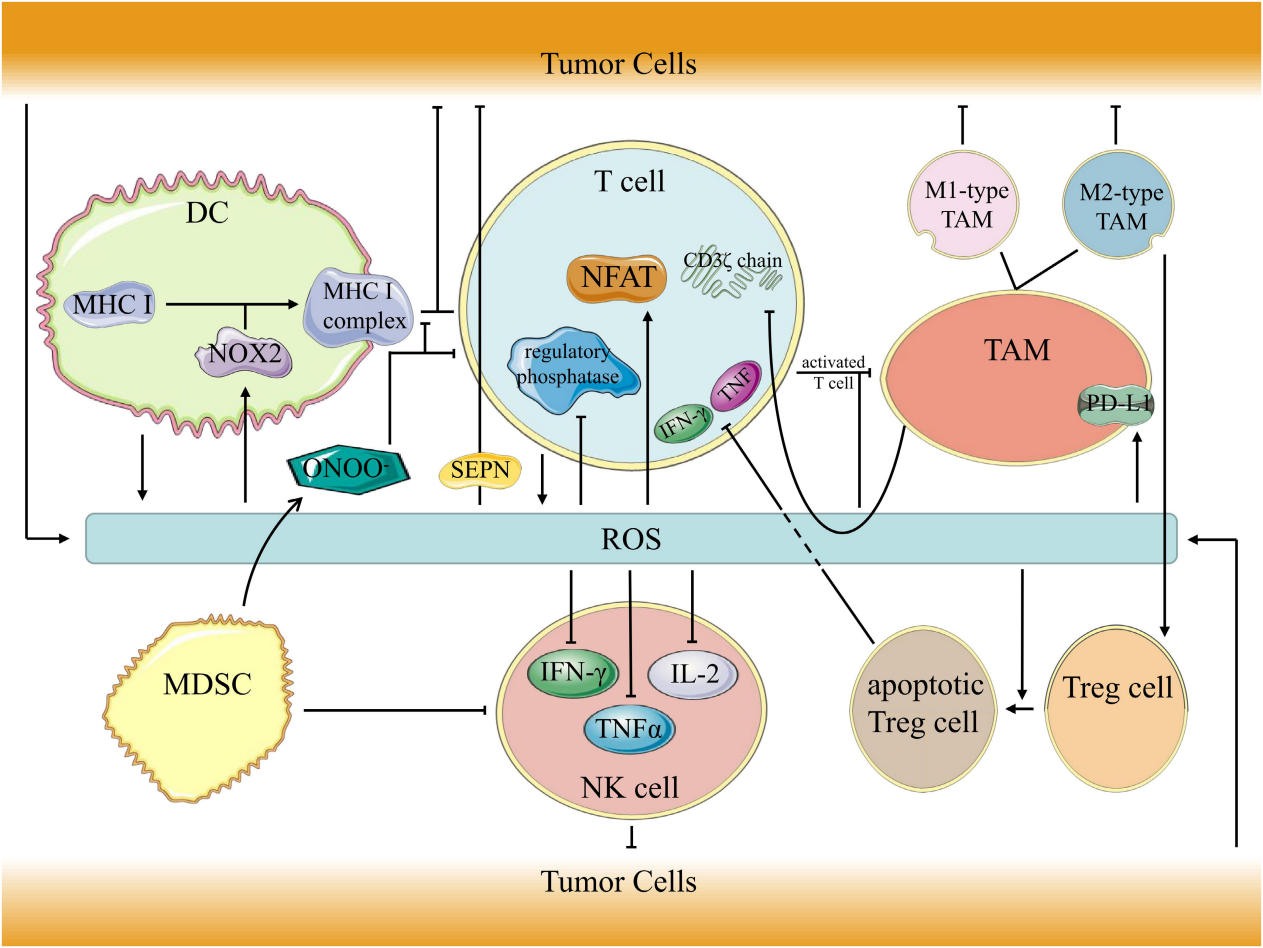
Effect of ROS on immune cells in tumor microenvironment.

Oxygen has a dual role in the TME and is not only involved in hypoxia. There is evidence that oxidative stress also has a profound effect on immune cells in the TME (Figure 1).^109^ Appropriate levels of ROS are necessary for antigen‐presenting cells to maintain normal function.^110^ Macrophages and dendritic cells (DCs) have been found to regulate antigen crosspresentation by producing phagosomal ROS in a process mediated by NOX2.^111^ The antigen presentation function of DCs is also believed to exist in PitNETs.^112^ Researchers stained macrophages with CD68 to study PitNETs. CD68^+^ cells were detected in all cases, and their number was positively correlated with tumor size and Knosp grade.^15^, ^113^ In addition, endoplasmic reticulum (ER) stress has an immunosuppressive effect and can lead to an increase in DCs and immunosuppressive signaling molecules, including arginase, which is related to antigen presentation disorders.^23^, ^114^, ^115^ In addition, ER oxidative stress induces immunogenic death of cancer cells through additional pathways.^110^, ^116^, ^117^, ^118^ Immunogenic cell death (ICD) leads to the release of damage‐associated molecular patterns (DAMPs) that bind to receptors on DCs and activate antigen cross‐presentation by DCs, ultimately generating an antitumor CD8^+^ T‐cell response.^110^, ^119^ Such effects provide a variety of viable options for regulating antigen presentation via ROS.

As one of the multiple immune cells in the TME, regulatory T cells (Tregs) suppress immune responses via oxidative stress.^120^, ^121^, ^122^ Increased Treg numbers and function are characteristics of PitNET patients and can be reversed by surgical resection of the tumor.^123^ On the one hand, Tregs are susceptible to oxidative stress‐induced apoptosis due to their weak Nrf2‐associated antioxidant system and susceptibility to free oxygen in the TME.^124^ On the other hand, apoptotic Tregs can inhibit the expression of interferon‐γ (IFN‐γ) and tumor necrosis factor (TNF) in CD4^+^ and CD8^+^ T cells, and this inhibitory effect is even stronger than that of live Tregs. Related studies have confirmed that apoptotic Tregs promote tumor growth more than live Tregs and are even able to nullify the antitumor effect of PD‐L1 blockade. The inhibitory effect of IFN‐γ on the development of PitNETs has been confirmed by single‐cell sequencing of PIT1‐positive PitNETs.^125^ In addition, immunosuppression by Tregs is somewhat dependent on macrophage function.^124^ Tumor Tregs were found to have higher mitochondrial activity and produce more endogenous ROS than conventional T cells, and these features make Tregs more sensitive to oxidative stress in the TME and consequently undergo apoptosis.^124^

Activation of T cells and NK cells leads to an increase in ROS, and conversely, ROS are important for T‐cell activation, expansion and effector functions.^126^, ^127^, ^128^, ^129^, ^130^ In addition, ROS, while contributing to T‐cell activation‐induced cell death, are also secondary messengers for nuclear factor of activated T cells (NFAT) activation and inhibition of negative regulatory phosphatases.^110^, ^127^ Increased ROS levels also trigger anti‐glutathione (GSH) responses that affect the metabolic integration and reprogramming of inflammatory T cells.^131^ Thus, it appears that oxidative stress leads to impaired T‐cell antitumor function, while ROS neutralization slows T‐cell terminal differentiation.^132^, ^133^ A study of somatotroph PitNETs revealed that the number of CD8^+^ lymphocytes was decreased in the cavernous sinus invasion group, suggesting that this factor may be able to predict the prognosis of somatotroph PitNETs.^134^ However, while high levels of ROS damage T cells, ROS are also necessary for T cells. Reactive oxygen species were found to maintain the metabolic and functional status of CD8^+^ T cells through SEPN family members in some tumors, thus enhancing the antitumor function of CD8^+^ T cells.^129^, ^135^, ^136^, ^137^, ^138^

The ability of NK cells to distinguish between malignant and healthy cells and their antigen‐independent tumor cell killing mechanism makes them an interesting tool for tumor therapy.^139^, ^140^ NK cells, when activated, tend to kill tumor cells and secrete a series of cytokines and chemokines that activate and recruit adaptive immune cells to the TME to induce antitumor effects.^141^, ^142^, ^143^ In addition to their cytotoxic function, NK cells also have a dual regulatory effect on antigen‐presenting cells (APCs).^144^, ^145^, ^146^ Overall, the TME promotes tumor growth and inhibits NK cells. NK cells use glucose as an energy source to generate the antitumor response.^147^, ^148^ A study found that NK cells in the TME had significantly increased expression of lipid peroxidation‐ and oxidative damage‐related proteins, which was related to NK cell dysfunction.^148^ In contrast, expanded NK (exNK) cells with metabolic adaptations similar to those of tumor cells had a greater capacity for nutrient uptake and glutamine export and were also able to survive better in the TME.^148^, ^149^, ^150^ In addition, excess ROS in NK cells blocks NF‐kB activation, leading to insufficient production of TNF‐α, IFN‐γ, and IL‐2.^151^, ^152^ These manifestations are similar to those of Tregs, and in fact, NK cells do have a very close association with T cells in oxidative stress‐related pathways. NK cell infiltration has been recognized as an indicator of PitNET progression. In one study, the researchers suggested that this has important implications for the development of immunotherapy for PitNETs.^153^

#### 4.2.2 Regulatory relationship between ROS and PD‐L1: effects on immune cells

PD‐L1 is a ligand of PD‐1, and the expression of PD‐L1 on the surface of cancer cells can inhibit the activation of T lymphocytes, and binding to PD‐1 can inhibit the escape of cytotoxic T cells.^154^ Studies have confirmed that PD‐L1 is significantly increased in metastatic PitNETs.^155^

In addition, the PD pathway also negatively affects DCs, TAMs, and NK cells, but the mechanism of PD‐1‐mediated inhibition is very complex, and there can be dual effects.^156^, ^157^, ^158^, ^159^, ^160^ Many factors are involved in the regulation of ROS and PD‐L1, among which NF‐kB, HIF‐1α and Yes‐associated protein 1 (YAP1) are some of the more critical.^161^, ^162^ HIF‐1α is a cellular oxygen sensor that is upregulated together with ROS under hypoxic conditions. Typically, activation of HIF‐α leads to upregulation of PD‐L1, which in turn leads to elevation of the expression of PD‐L1 on the cancer cell surface.^154^ The regulation of PD‐L1 and HIF‐1α is bidirectional. PD‐L1 induces upregulation of HIF‐1α through ROS production, thereby upregulating YAP1 expression in cancer cells.^161^ In addition, NF‐kB also contributes to the regulatory relationship between ROS and PD‐L1. Subsequently, MDSCs, Tregs, and TAMs infiltrate tumors, which in turn stimulates PD‐L1 expression in tumor cells.^154^ The increase in ROS is often due to mitochondrial dysfunction, which has been demonstrated in PitNET cells.^66^ This implies that the regulatory relationship between ROS and PD‐L1 may be present in metastatic PitNETs, representing a new therapeutic target.

## 5 TARGETING OXIDATIVE STRESS TO TREAT PITNETS

### 5.1 Induction of oxidative stress in PitNETs

Mitochondria have been mentioned previously as the source of endogenous ROS production. Therefore, there have been several studies targeting mitochondrial oxidative stress for the treatment of PitNETs (Table 1), some of which have identified feasible strategies.^163^, ^164^

**TABLE 1:** Drugs targeting mitochondria to treat PitNETs.

Drug	Mechanism	Experimental results	Species	Reference
18β‐glycyrrhetinic acid	Increase of lactate dehydrogenase release and increase of ROS and Ca^2+^ concentration	Decreased activity of pituitary adenoma‐derived MMQ and GH3 cells in rats	Rat	Wang et al.^169^
	Inhibition of mitochondrial membrane potential and downregulation of the ratio of Bcl‐2 and Bax	Cell cycle arrest, increased apoptosis rate and increased mitochondrial membrane permeability		
	Activation of calcium/calmodulin dependent protein kinase II (CaMKII), JNK and P38	Significantly inhibit the growth of pituitary adenoma in nude mice		
Cyclosporine A (CsA)	Nuclear and DNA fragmentation and increased p53 expression	Apparent death of apoptotic cells	Rat	Kim et al.^182^
	Bax level increased in a dose‐dependent manner in two types of cell death, while Bcl‐2 level increased in a dose‐dependent manner in autophagy and decreased in a dose‐dependent manner in apoptosis	Induce of apoptotic or autophagic cell death in rat pituitary GH3 cells		
Dopamine agonists	Bromocriptine induces the apoptosis of prolactinoma cells through the ERK/EGR1 signaling pathway	Bromocriptine induced prolactinoma cell death mainly through the apoptosis pathway	Rat	Tang et al.^189^
	Cabergoline induces autophagic death by inhibiting the AKT/mTOR signaling pathway	Cabergoline induced pituitary prolactinoma cell death mainly via the autophagic cell death pathway		
	Bromocriptine treatment reduced the tumor size of GH3 xenotransplantation model in nude mice	GH3 cells are more sensitive to Bromocriptine	Nude mice	
Gossypol acetate	The expression of miR‐15a was upregulated and the expression of Bcl‐2 was decreased	Induce the morphological changes of mitochondria, cause tumor cell apoptosis, and inhibit tumor growth	Rat	Tang et al.^192^
Melatonin	Inhibition of mitochondrial respiratory complex activity and ATP production in prolactinoma cells	Regulate mitochondrial function and energy metabolism of prolactinoma cells	Rat	Wang et al.^206^
	Inhibition of Bcl‐2 mRNA expression and mitochondrial membrane potential			
Paeoniflorin	Enhance the expression of cleave caspase‐9, ‐3 and Bax and inhibit the expression of Bcl‐2 in MMQ cells, thereby inducing mitochondria‐mediated apoptosis	Decrease of MMQ and GH3 cell viability and inhibition of cell proliferation	Rat	Wei et al.^195^
Liquiritigenin	Inhibited cell viability, caused G1 phase arrest and initiated apoptosis in both MMQ and GH3 cells, increased intracellular ROS level and cytosol cytochrome C	Inhibits PitNET growth and induces cell apoptotic death mainly via Ras/ERKs and ROS‐dependent mitochondrial pathways	Rat	Wang et al.^163^
		Markedly reduced tumor size without affecting bodyweight in mice with GH3 cells xenograft	Mice	
Artesunate	Induced G0/G1 phase arrest, accumulated in the mitochondria of MMQ cells, inhibiting mitochondrial respiratory function and mediating apoptosis through the mitochondrial pathway	Specifically inhibited MMQ proliferation and PRL synthesis and activated the apoptosis of MMQ cell	Rat	Zhang et al.^164^
		Inhibited proliferation and activated the apoptosis of MMQ cells	Mice	

18β‐Glycyrrhetinic acid is a natural compound extracted from liquorice that has been shown to have anticancer and anti‐inflammatory effects.^165^, ^166^, ^167^, ^168^ Administration of 18β‐glycyrrhetinic acid to rat pituitary adenoma‐derived MMQ and GH3 cells resulted in increased lactate dehydrogenase release, increased intracellular ROS and Ca^2+^ concentrations, and reduced cell viability.^169^ 18β‐Glycyrrhetinic acid induced altered mitochondrial membrane permeability and mitochondrial dysfunction, leading to activation of cytochrome C and caspase‐3 and inhibition of Bcl2 expression, ultimately inducing apoptosis of PitNET cells.^167^, ^169^ In addition, intracellular ROS levels increased significantly after 12 h in the presence of 18β‐glycyrrhetinic acid. Furthermore, the decrease in cell viability induced by 18β‐glycyrrhetinic acid was significantly counteracted by pretreatment with NAC (an ROS inhibitor), indicating that the proapoptotic effect of 18β‐glycyrrhetinic acid was promoted by ROS.^169^ The mitogen‐activated protein kinase (MAPK) cascade is a key signaling pathway regulating a variety of cellular processes, including proliferation, differentiation, apoptosis, and stress responses.^170^ Members of the c‐Jun N‐terminal kinase (JNK) subfamily play an important role in cellular oxidative stress and apoptosis.^171^ 18β‐Glycyrrhetinic acid enhanced the phosphorylation of JNK and p38 at 0.5 h, and reversal of JNK and p38 phosphorylation significantly attenuated the decreased cell viability induced by 18β‐glycyrrhetinic acid.^169^, ^172^, ^173^, ^174^ Moreover, the activation of JNK and p38 induced by 18β‐glycyrrhetinic acid was almost completely inhibited by NAC pretreatment, indicating that NAC pretreatment induces phosphorylation of JNK and p38 by causing the accumulation of ROS in PitNET cells to exert cytotoxic effects.^169^

Cyclosporine A (CsA) is a widely used immunosuppressant that induces both cellular autophagy and apoptosis.^175^, ^176^, ^177^ Recent studies have found that CsA induces ER stress, increases mitochondrial ROS production, disrupts redox homeostasis, and triggers lipid peroxidation.^178^, ^179^, ^180^, ^181^ Researchers found that the expression of intracellular antioxidant enzymes such as superoxide dismutase (SOD), catalase and GPx was decreased with CsA‐related toxicity. Therefore, the Cu/Zn‐SOD level was measured in CsA‐treated GH3 cells and decreased after high‐dose CsA treatment. Similarly, the Mn‐SOD level also decreased in a dose‐dependent manner, potentially revealing one of the pathways by which CsA leads to GH3 cell apoptosis.^182^ In addition, similar to 18β‐glycyrrhetinic acid, CsA was able to decrease mitochondrial membrane potential and alter mitochondrial membrane permeability, which in turn caused the release of cytochrome C and ultimately inhibited Bcl2 expression and induced apoptosis.^183^ Interestingly, in studies on CsA nephrotoxicity, ERK, JNK, p38 and NF‐kB were found to be targets of CsA, and CsA was also found to strongly activate Nrf2 in proximal tubular epithelial cells, which, as previously mentioned, are key factors in the induction of oxidative stress.^181^, ^184^, ^185^, ^186^ In addition, CsA has been found to directly trigger a decrease in antioxidant capacity in a variety of tissue cells.^187^, ^188^ This mechanism renders cellular redox homeostasis unstable and makes cells more susceptible to oxidative stress. These findings suggest that CsA may have unprecedented potential in inducing oxidative stress in PitNET cells.^182^

Numerous other drugs have also been studied. Dopamine agonists, mainly bromocriptine (BRC) and cabergoline (CAB), are effective in reducing the size of prolactinomas. BRC induces apoptosis of prolactinoma cells primarily through the ERK/EGR1 signaling pathway, while CAB induces autophagy by inhibiting the AKT/mTOR signaling pathway.^189^, ^190^, ^191^ Gossypol acetate (GAA) has the ability to inhibit enzymatic activity, causing changes in mitochondrial morphology and inhibiting Bcl2 expression, thereby inducing apoptosis.^192^ Melatonin is able to selectively block the production of VEGF and ROS and even reverse angiogenesis, which can result in reduced ROS and a lack of tumor nutrition in the TME.^193^ Paeoniflorin increased the expression of cleaved caspase‐9, caspase‐3, and Bax and inhibited the expression of Bcl‐2 in MMQ cells and GH3 cells, thereby inducing mitochondria‐mediated apoptosis, which ultimately manifested as a decrease in cell viability and inhibited proliferation.^194^, ^195^

### 5.2 Immunotherapies for PitNET targeting tumor redox

The human immune system achieves tumor surveillance under normal conditions through innate and adaptive immune responses against tumor antigens. However, tumor cells can evade monitoring by the immune system and further proliferate, infiltrate, and metastasize.^155^ Therefore, immunotherapies have been developed to activate immune cells in the TME to enable them to generate an immune response and ultimately inhibit tumor growth. TNF and IL‐2 were explored in early studies, but they are no longer used for routine treatment due to unacceptable systemic side effects.^196^ In the quest for additional immunotherapies, strategies targeting immune cells in the TME and modulating oxidative stress pathways are emerging due to the abnormal ROS levels in tumor cells.

#### 5.2.1 Melatonin

Melatonin is a natural hormone secreted mainly by the pineal gland. Melatonin has antioxidant effects because it reacts directly with free radicals and induces the production of antioxidant enzymes.^197^, ^198^ Correspondingly, increased melatonin release at night reduces oxidative DNA damage, and conversely, oxidative DNA damage is increased during the day.^199^, ^200^, ^201^ The inhibition of indoleamine 2,3‐dioxygenase 1 (IDO1) induced by melatonin is associated with the activation and maturation of DCs and activates cytotoxic CD8^+^ T lymphocytes.^201^, ^202^, ^203^, ^204^ In addition, investigators found a dose‐dependent decrease in ROS levels in melatonin‐treated CD4^+^ T cells.^205^ This suggests that melatonin has the potential to rescue CD4^+^ T cells that die in the TME due to excessive ROS levels. Studies on prolactinoma showed that melatonin inhibited the activity of the mitochondrial respiratory complex and ATP production in prolactinoma cells.^206^ Another study found that melatonin can decrease Bcl‐2 mRNA expression and mitochondrial membrane potential.^207^ These effects enable melatonin to regulate the mitochondrial function and energy metabolism of prolactinoma cells.^206^, ^207^ In the previous section, we mentioned the ability of melatonin to inhibit angiogenesis, and here, we have reported the additional ability of melatonin to activate immune cells in the TME. Such dual effects suggest that melatonin has potential in the treatment of PitNETs, and in vitro tests have demonstrated that melatonin inhibits the growth of prolactin tumor cells in rats.^197^

#### 5.2.2 Phenformin

Immunotherapy targeting PD‐L1 has been well‐studied, and compounds affecting PD‐L1 via ROS have been discovered (Table 2). Although these compounds have the potential to be used in the treatment of PitNETs, few compounds have been investigated in PitNETs.^208^, ^209^, ^210^, ^211^, ^212^, ^213^, ^214^ Phenformin is a biguanide that is used as an antidiabetic agent. In addition, phenformin has been found to have antitumor effects on several tumors.^215^ Phenformin is thought to exert antitumor effects through oxidative stress pathways, as evidenced by decreased GSH expression and a reduction in the GSH reduction ratio.^216^, ^217^ Furthermore, phenformin induces ROS production in MDSCs.^215^ In addition, phenformin also inhibits mTOR phosphorylation and HIF‐1α expression, which attenuates PD‐L1 expression.^154^, ^217^ Surprisingly, these effects did not attenuate the effect of anti‐PD‐L1 treatment, and combination therapy administration synergistically induced CD8^+^ T‐cell infiltration and reduced the levels of key proteins affecting the immunosuppressive activity of MDSCs.^215^ Experiments with PitNET cell lines showed that biguanides reduced the viability and increased the apoptosis of all PitNET cells and cell lines, with phenformin having the most potent effects.^218^ This result validates the feasibility of phenformin as a PitNET treatment and reveals its impressive potential.

**TABLE 2:** Compounds affecting PD‐L1 via ROS.

Compounds	Mechanism	Effect	Animal models/cell lines	Reference
Phenformin	Selectively inhibits granulocytic MDSCs in spleens of tumor‐bearing mice and ex vivo, induces production of reactive oxygen species in granulocytic MDSCs	Combination of phenformin and anti PD‐1 cooperatively induces CD8^+^ T cell infiltration, and enhances the effect of anti‐PD‐1 antibody therapy on inhibiting tumor growth	Melanoma mouse model	Kim et al.^215^
Anlotinib	Activates ROS/JNK/activator proton‐1 (AP‐1) signal pathway, increase the percentage of NK cells and M1‐TAMs in TME, and reduce the percentage of M2‐TAMs	Improves the expression level of PD‐L1	Mouse and human colorectal cancer (CRC) cell lines	Luo et al.^208^
pep‐PAPM@PTX^a^	Binds the cell surface PD‐L1 and drives its recycling to lysosomal degradation, thus reverting PTX‐induced PD‐L1 upregulation and downregulating PD‐L1 expression	Significantly promoted T cell infiltration and increased tumor immunoactivating factors, achieve enhanced anticancer potency	Triple‐negative breast cancer model	Hu et al.^209^
IR775@Met@Lip^b^	Reverses tumor hypoxia to enhance ROS production to elicit more chemical damage	Successfully suppressed both the primary and abscopal tumor growth in bladder and colon cancers, respectively	Murine bladder tumor cells and murine colorectal tumor cells; male mice	Xiong et al.^210^
Photodynamic therapy (PDT)	Effectively downregulates the overexpression of PD‐L1			
Ethaselen	Inhibition of thioredoxin reductase activity, elevation of ROS, decrease of EGFR activation, significantly reduce PD‐L1 level	Suppress the progression of primary tumor and eliminate tumor metastasis	Human breast cancer cell line and CRC cell line; mouse breast cancer 4T1 model	Zheng et al.^211^
Trifluoperazine	Decreased mitochondrial membrane potential, and increased ROS level	Induce G0/G1 cell cycle arrest, significantly inhibit cancer cell proliferation and tumor growth	Human CRC cell line, mouse CRC cell line; mice CRC subcutaneous tumor models	Xia et al.^212^
	Increased the expression levels of PD‐L1 in CRC cells and PD‐1 in tumor‐infiltrating CD4^+^ and CD8^+^ T cells			
Ferric ammonium citrate	Increase ROS production in macrophages and upregulate PD‐L1 expression	Altered the phenotype of macrophages, resulting in the enrichment of immunomodulatory T cell subsets	Mice	Choi et al.^213^
Pemetrexed	Increase ROS level, activate NF‐κB, and then upregulate PD‐L1 expression	Combined treatment with PD‐L1 creates a more favorable immunotherapy TME	Human lung cancer cell lines and the human breast cancer cell line	Lu et al.^214^

^a^ pep‐PAPM@PTX: Integrating physically‐encapsulated paclitaxel (PTX) and surface‐modified anti‐PD‐L1 peptide (pep) for combined chemotherapy.

^b^ IR775@Met@Lip: A composite by loading metformin and IR775 into a clinically usable liposome as a two‐in‐one nanoplatform.

#### 5.2.3 Baicalein

Baicalein is a flavonoid compound extracted from the roots of *Scutellaria baicalensis* and is used as an antioxidant, anti‐inflammatory and antiviral treatment.^219^, ^220^, ^221^, ^222^ In recent years, an increasing number of studies have shown that baicalein inhibits tumor cell proliferation and metastasis by inducing apoptosis and senescence of cancer cells.^223^, ^224^ Baicalein was found to affect the Nrf2/HO‐1 signaling pathway, inhibit NF‐kB phosphorylation and activate Nrf2, thereby decreasing the abnormally increased ROS levels in MDSCs.^225^ This inhibition of MDSCs relieves the inhibition of other immune cells. Toll‐like receptor 4 (TLR4) is thought to mediate the proliferation of primary PitNET cells.^226^ Coincidentally, TLR4 is inhibited by baicalein, and the expression of its downstream signaling factor HIF‐1α is also reduced.^227^ As we mentioned, the reduction in HIF‐1α expression causes PD‐L1 expression to be reduced.^154^ In addition, baicalein significantly inhibited IFN‐γ‐induced PD‐L1 upregulation,^228^ and baicalein inhibited the expression of VEGF.^227^ These results suggest that baicalein may be a target for PD‐L1‐related PitNET immunotherapy.

## 6 CONCLUSIONS AND PERSPECTIVES

Although there are many treatment options available for PitNETs, effective treatment options for refractory and metastatic PitNETs are still very limited. In recent years, immunotherapy for tumors has gained more research attention, with unexpected results in the treatment of many tumors. Therefore, immunotherapy has likewise been attempted for the treatment of PitNETs. To identify additional therapy targets and achieve better results, oxidative stress has been considered. Oxidative stress is of great importance in both tumors and the TME, mediating numerous biochemical reactions. The potential of strategies related to oxidative stress in the treatment of PitNETs has revealed many new research directions. On one hand, oxidative stress can act directly on tumor cells to inhibit tumor cell proliferation and metastasis. However, oxidative stress can act on immune cells in the TME and mediate the immunotherapy response.

Popular targets of immunotherapy include PD‐L1 and cytotoxic T lymphocyte‐associated antigen 4 (CTLA4), but CTLA4 has been little studied in PitNETs, so we focused on PD‐L1.^229^ Tumor‐mediated metabolic disorders are the main drivers of immune dysfunction, and oxidative stress plays an important role in this process. Regarding immune cells, ROS are involved in antigen presentation and activate or inhibit different immune‐related cells. Regarding tumor cells, ROS mediate the immunotherapy response by affecting PD‐L1 expression on the surface of tumor cells, in addition to directly mediating their apoptosis. The use of anti‐PD‐L1 antibodies for the treatment of PitNETs has been reported for a few cases, but most of them have been isolated.^155^ Some compounds targeting ROS have shown synergistic effects with anti‐PD‐L1 therapy in trials.

In addition to the compounds mentioned in this review, some emerging ROS‐modulating agents have been suggested to modulate the response of tumors to immunotherapy.^110^ High‐dose ascorbate showed a synergistic effect with anti‐PD‐1 treatment in mouse tumor models and was able to enhance CD8^+^ T‐cell infiltration, and this effect seemed to be independent of its antioxidant capacity.^230^, ^231^ Nonsteroidal anti‐inflammatory drugs (NSAIDs), including celecoxib and aspirin, have also been reported to stimulate antitumor immune responses, and enhance the efficacy of anti‐PD‐1 treatment in mouse tumor models.^232^, ^233^, ^234^ In addition, xCT inhibitors and ROS‐response prodrugs have been suggested to affect the immunotherapy response of tumors.^110^

In summary, oxidative stress mediates antitumor therapy by acting on PitNETs themselves and immune cells in the TME. This emerging therapeutic pathway has surprising potential and remains to be investigated further.

## AUTHOR CONTRIBUTIONS

YZ, AZ, and CF designed the review and wrote the manuscript. CF and LY conceived the artwork and performed the bibliographical research. AS, YX, and DZ supervised the writing. All the authors revised and approved the final version of the manuscript.

## FUNDING INFORMATION

This work was supported by grants from the Project Supported by Zhejiang Provincial Natural Science Foundation of China (LY22H090020) and Shanghai Pujiang Program (22PJD009).

## CONFLICT OF INTEREST STATEMENT

The authors declare no conflict of interest.

## Data Availability

The data that support the findings of this study are available from the corresponding author upon reasonable request.
